# Indirect self-presentation of people with machiavellianism accentuation

**DOI:** 10.1192/j.eurpsy.2021.1181

**Published:** 2021-08-13

**Authors:** T. Folomeeva, S. Fedotova, I. Beloglazov

**Affiliations:** The Department Of Psychology, Lomonosov Moscow State University, Moscow, Russian Federation

**Keywords:** Indirect Self-Presentation, Machiavellianism, Self-Presentation

## Abstract

**Introduction:**

Personal traits influence persons’ perception of the social environment. Therefore analyzing stories with the non-specific plot can enable to distinguish particular characteristics.

**Objectives:**

The aim is to determine the features of verbal self-presentation of people with high and low scores on the Machiavellian scale.

**Methods:**

1. For the selection of particular participants, who have high and low scores, the questionnaire «Dark triad» of Egorova was used. 2. For collection stories of respondents, a series of interviews was carried out with extra stimulus. There were 20 conversations. Age was from 19 to 29 (m = 23; sd = 7,1).

**Results:**

The opportunity to predict personal traits in general stories was proved. There is a confrontation between the person and the world in the speech of the Machiavellians. Their stories usually have a strong hero, other characters are ignored by the main person. Machiavellians want a safe and calm place that allows them to be themselves. We assume that this is a consequence of the fact that they have to dissemble in society. This statement requires further verification Non-Machiavellians are concerned by the opinion of society, that affects their life and behavior. They act for the well-being of the world while their own feelings are being ignored. There is a feeling of guilty in non-Machiavellians’ tales which is connected with failures around them.

**Conclusions:**

The study was piloted interviewing method (with stimulus material) for the study of indirect verbal self-presentation. Differences were found between the people’s self-presentation with an accentuation of Machiavellianism and non-Machiavellianism.

